# Stoichiometry Based Steady-State Hepatic Flux Analysis: Computational and Experimental Aspects

**DOI:** 10.3390/metabo2010268

**Published:** 2012-03-14

**Authors:** Mehmet A. Orman, John Mattick, Ioannis P. Androulakis, Francois Berthiaume, Marianthi G. Ierapetritou

**Affiliations:** 1 Department of Chemical and Biochemical Engineering, Rutgers, the State University of New Jersey, Piscataway, NJ 08854, USA; 2 Department of Biomedical Engineering, Rutgers, the State University of New Jersey, Piscataway, NJ 08854, USA

**Keywords:** hepatocytes, perfused livers, metabolic flux analysis, flux balance analysis, metabolic pathway analysis

## Abstract

: The liver has many complex physiological functions, including lipid, protein and carbohydrate metabolism, as well as bile and urea production. It detoxifies toxic substances and medicinal products. It also plays a key role in the onset and maintenance of abnormal metabolic patterns associated with various disease states, such as burns, infections and major traumas. Liver cells have been commonly used in *in vitro* experiments to elucidate the toxic effects of drugs and metabolic changes caused by aberrant metabolic conditions, and to improve the functions of existing systems, such as bioartificial liver. More recently, isolated liver perfusion systems have been increasingly used to characterize intrinsic metabolic changes in the liver caused by various perturbations, including systemic injury, hepatotoxin exposure and warm ischemia. Metabolic engineering tools have been widely applied to these systems to identify metabolic flux distributions using metabolic flux analysis or flux balance analysis and to characterize the topology of the networks using metabolic pathway analysis. In this context, hepatic metabolic models, together with experimental methodologies where hepatocytes or perfused livers are mainly investigated, are described in detail in this review. The challenges and opportunities are also discussed extensively.

## 1. Introduction

The liver has many complex physiological functions. It plays an important role during the hyper-metabolic state associated with various diseases, including burns, infections and major trauma that are characterized by an accelerated breakdown of skeletal muscle protein, increased resting energy expenditure and a negative nitrogen balance. Isolated perfused livers have been commonly used to characterize disease-related metabolic alterations at the organ level [1,2,3,4,5,6,7]. Liver cells, mainly hepatocytes, have been also used *in vitro* experiments to elucidate the effects of drugs and metabolic changes caused by specific conditions. These experiments performed at cell and/or organ levels are important for functional characterization of the mammalian systems or to improve the functions of medicinally important tools.

There is a significant number of studies in literature where metabolic properties of the liver have been investigated. Herein, we mainly focus on the studies where metabolic network of hepatocytes or the organ itself have been investigated, using metabolic engineering tools such as metabolic flux analysis, flux balance analysis and pathway analysis. After briefly summarizing the functions of the liver, this review paper explains the experimental procedures and the stoichiometric models where perfused livers or hepatocytes have been utilized. Major outcomes of these studies and current challenges are also comprehensively discussed.

## 2. Functions of the Liver

The liver is an extremely important organ that has a variety of roles in mammalian function, including blood protein synthesis, metabolism of nutrients, removal of toxic substances and the synthesis of bile. It is unique amongst mammalian organs due to its ability to regenerate [8]. It is also involved in the mammalian systemic response to infection and injuries. Hepatocyte behavior is strongly altered by the presence of bacteria, and liver failure is often one of the symptoms of chronic sepsis. The liver comprises approximately 70% hepatocytes, which are the cells that serve the primary functions of the liver, and 20% Kupffer cells, which are macrophages that permanently reside in the liver, and serve to defend it from infection and promote an inflammatory response during innate immunity [9].

As a regulator of metabolism, the liver is heavily involved in the synthesis of glycogen, fatty acid production and consumption, and the production of glucose. Glucose production is performed through a series of metabolic reactions that convert pyruvate to glucose, known as gluconeogenesis. The glucose that is produced by the liver is exported out to other cells in the body, and is utilized either through aerobic or anaerobic respiration. The liver plays a crucial role in the Cori cycle. This is the process whereby muscles produce lactate via glycolysis, which is then taken up by the liver and converted back into glucose via gluconeogenesis. Glucose is then transported back to the muscle cells, thereby replenishing their energy stores [10]. Glycogen is a polymer created from glucose in the liver, and it is one of the key indicators of energetic health in the liver. The dysfunction of glycogen regulation has serious repercussions in the liver [11], and thus its storage, synthesis and breakdown are tightly regulated by the organ. The liver has also been strongly associated with amino acid production, degradation and utilization. The supplementation of various amino acids has been shown to have a positive effect on liver function, including a decrease in protein degradation [12]. The production of cholesterol, which is an important small molecule that governs the distribution of lipids throughout the body, cell membrane permeability and other important properties of cells, is controlled by the liver. The liver has been implicated in the production of fibrinogen, albumin and globulin proteins. These proteins are critical for clot formation, wound healing and immune system function.

Another major role that the liver has in mammalian organ function is that it detoxifies a variety of foreign objects in the blood stream through a process called drug metabolism, which makes up a variety of chemical modifications that can be applied to foreign substances to make them prone to further metabolism [13]. One of the mechanisms that the liver employs to accomplish this is done through cytochrome p450, which is a family of mitochondrial enzymes that provides reactive oxygen species that can interact with any chemical compound to oxidize it [14], thereby potentially making it more easily metabolized. However, this can have drawbacks, as the production of these oxygen species can cause damage to the mitochondria and impair liver function [15]. Another important cycle that takes place in liver is the urea cycle, which is used to remove the toxic product, ammonia, from the body. This is done by liver by converting carbon dioxide and ammonia into urea, for the purposes of excretion of the urea through the urinary tract. In pathophysiology, dysfunction of the cycle can cause hypertension issues [16], as well as nitrogen loss during systemic inflammation [17].

Liver cells can exhibit different functions based on their location (hepatic zonation). This phenomenon is caused by heterogeneity within the liver that arises from differences in blood supply: hepatocytes that are located in the upstream zone of the liver contain different enzymes and receptors than their downstream counterparts, leading to highly specialized metabolic capabilities [18]. The hepatic zonation can be divided into two basic categories: Periportal cells, which are located in close proximity to the portal vein that exits the liver, and perivenous cells, which are located in close proximity to the central vein that enters the liver [19]. These divisions can be further divided into proximal and distal portions [20]. However, these distinctions are less pronounced compared to the differences between periportal and perivenous hepatocytes. Hepatocytes in the periportal zone are characterized by their up-regulation of oxidative phosphorylation [21], glucose production [22], urea formation [23], anti oxidant metabolism, plasma protein synthesis (albumin, α2-macroglobulin, fibrinogen), cholesterol synthesis and bile formation [24]. In contrast, perivenous hepatocytes are characterized by their up-regulation of glucose uptake [25], glutamine formation [26], xenobiotic metabolism, fatty acid synthesis [27] and plasma protein synthesis (a-Fetoprotein, angiotensinogen, and α1-antitrypsin) [24].

## 3. Experimental Methods for Liver Systems

### 3.1. Liver Cell Isolation and Culturing

Using primary hepatocytes for the analysis of hepatic metabolism has been a widely used practice that allows investigators to observe liver cell function and metabolism in a highly controlled environment. In order to prepare this model system, it is necessary to first isolate hepatocytes from the liver, which has been well characterized by perfusion with collagenase [28]. In this method, the liver is cannulated at the portal vein and the intra hepatic vein, and collagenase is run through the system, breaking down the extracellular matrix and allowing hepatocytes to break free from the liver [29]. The surgery and isolation is well characterized by many studies, and has become a kind of gold standard for rat hepatocyte isolation in *in vitro* metabolic studies. This process is not unique to rats, and has been performed on many other mammalian species, making it ideal for assessing liver function in a broad variety of mammalian systems [30]. Assessing hepatocyte viability is an important part of this isolation process, since the loss of this viability can have serious consequences on the metabolic pathways being analyzed within the cell [31]. Another issue with hepatocyte isolation is the introduction of other cell types, which can include Kupffer cells and endothelial cells that were also dislodged by the use of collagenase [32]. The introduction of these other cell types can impact on hepatocyte growth, viability, and behavior, and it is up to the researcher to decide if those cell types should be purified or kept [33]. Once the cells have been adequately purified, the standard plating method for hepatocytes is to culture the cells on a collagen base [34]. Collagen is the ideal substrate, since the cells are anchored on collagen *in vivo*, and without it they cannot form the extracellular structures necessary for both viability and function [35]. Traditionally, only a single layer of collagen has been used in cell culture similar to many other cell types [36]. However, the physical structure and layout of the collagen base has been significantly optimized and altered in recent years. Sharma *et al*. were able to create a more *in vivo*-like environment for hepatocytes to grow in, using an adipogel generated from the differentiation of preadipocytes [37]. The differentiation of preadipocytes generates an extracellular adipogel matrix which can be placed in the medium on top of a collagen base in order to create a three-dimensional extracellular matrix for hepatocytes to grow in. The authors show that this process is able to enhance the viability and metabolic function of hepatocytes, and create a more *in vivo*-like environment. Borel Rinkes *et al*. used sandwiched collagen layers in order to create a three-dimensional hepatocyte culture, where layers of hepatocytes were maintained between two layers of collagen, preserving long-term cell function in *in vitro* cultures [38]. This has the benefit of mimicking *in vivo* structure, and improving the diffusion of substrates to all hepatocytes simultaneously. This is important, as many metabolic studies aim to characterize the effects of specific substrates on hepatocyte function [39]. The other important consideration that an investigator must take into account when culturing hepatocytes is the composition of the medium. The composition of the medium can have a serious impact on the viability and metabolic function of hepatocytes, with physiological parameters such as pH and metabolite concentrations within the media having a significant impact on hepatocyte function [40]. Specific proteins that are commonly found in human serum also are important in hepatocyte media, and it is necessary to include the appropriate proportions of albumin, and salts [41]. Overall, cultures of hepatocytes are a very useful tool for the assessment of metabolic function: The amount of control that an investigator can exert over the system is significantly higher than the amount of experimental flexibility in *in vivo* systems, and the impact of individual substrates can easily be measured. However, care must be taken in the isolation, cell purity and medium quality of the culture in order to improve viability, and the collagen base should be optimized to create as *in vivo*-like conditions as possible.

### 3.2. Liver Perfusion System

Though primary hepatocytes isolated from the liver can provide important metabolic data, the culturing process removes the cells from their *in vivo* environment and prevents their exposure to a variety of extracellular processes. Because the dynamics of many of the diseases studied *in vitro* are not completely understood, it is difficult for investigators to completely mimic the *in vivo* conditions of these pathogenic processes. Isolated perfusion of rat livers is one method that has been extensively explored in recent years for assessing the metabolic function of the liver *in vivo* following an insult or injury [1]. The isolated perfused rat liver (IPRL) procedure has been described previously in detail [42]. IPRL is performed using a surgical technique that is very similar to the method in which collagenase is used to isolate hepatocytes: Rats are anesthetized and their abdominal cavities are exposed. Heparin might be injected by transphrenic cardiac puncture to prevent blood clotting throughout the surgical manipulations. Following this, the hepatic artery and the suprarenal vena cava are ligated, and the portal vein is cannulated to send perfusate to the liver. The liver outflow from the hepatic vein is collected through the catheter that is cannulated into the inferior vena cava via the right atrium. The liver is then flushed with medium for 10–20 minutes on an open circuit, and following this, the circuit is closed and the medium is collected. The liver is perfused *insitu* at a constant flow rate by a peristaltic pump. The perfusion system also includes a heat exchanger (to get a perfusate at physiological temperature), an oxygenator (to oxygenate the perfusate) and a bubble trap (to remove the bubbles in the perfusate which might block the blood vessels in the liver). One important consideration in this procedure is the flow rate at which the medium is passed through the liver: If the pressure is too high, liver trauma can occur, which significantly impacts liver viability and will compromise the effectiveness of the study [43]. Another important physical consideration is temperature: Since liver core temperature *in vivo* is at 37 °C, it is important to maintain that temperature throughout the liver during the perfusion. A variation in the temperature of more than 1 °C can cause a distortion of results, and impact liver function [44]. The duration of the perfusion is important as well, and a liver can only be safely perfused for up to 120 minutes, before results are observed to be significantly impacted [45]. One of the more controversial aspects of liver perfusion over the past 10 years has been the oxygenation of the liver. Traditionally, simply oxygenating the medium was considered to be sufficient in order to provide adequate oxygenation of the liver, since the oxygen carried by the medium would act in conjunction with diffused oxygen from the atmosphere into the exposed liver. Recently, however, this conclusion has come under scrutiny, as recent studies have shown that when the perfusate does not contain oxygen carriers, it cannot deliver sufficient oxygen to the liver and metabolism is impacted and results are skewed [46]. Although increased flow rates into the liver can help deliver more oxygen, the increased pressure from the flow can cause significant liver trauma, as described previously. This is an important aspect of metabolic function, since Mik *et al*. have shown that even a slight difference in mitochondrial oxygen concentration can significantly impact cellular function [47]. One method of providing oxygen in the liver has been to incorporate bovine red blood cells into the medium, and expose them to oxygen, allowing for a significantly higher level of oxygen to be delivered to the liver on a continuous basis [46,48]. Another method has been to incorporate emulsion-based oxygen carriers into the perfusion medium, which have a much higher oxygen saturation concentration, allowing for the delivery of oxygen to the liver without the introduction of foreign cells [49]. Similar to primary hepatocyte cultures, the appropriate selection of media is important for the accurate assessment of liver viability. The composition of the medium has been shown to impact the metabolic functions of the liver and alter metabolic rates in the liver [48]. It has been shown to impact the toxicity of certain compounds, and can alter the way in which the liver metabolizes xenobiotic substrates and other compounds relevant to drug toxicity assessments [50]. Beyond the medium composition that includes the proteins and salts that are native to serum [2], it is important to include metabolites that are of interest to the study being conducted. Experiments that are designed to assess metabolic networks in IPRLs must be sure to include all of the external metabolites in the perfusate, in order to assess both the uptake and production of each of these metabolites. Studies have been conducted that make strong use of computational methods and appropriate IPRL systems in order to assess the effects of injuries and nutritional supplements on liver metabolism and liver failure [3]. Other experiments include radio labeling experiments, which use radioactive substrates to directly assess the intracellular flux of specific pathways [5]. For radio labeling studies of protein synthesis and degradation, it can be necessary to prime the liver with amino acids over the washing period, in order to shift their behavior towards uptake through a concentration gradient [6]. IPRL remains an important experimental method for the assessment of metabolic pathways, which can be done through external flux analysis, radio labeling and pathway analysis, or with light and electron microscopy. In order to ensure that liver conditions do not impact results, it is important to standardize medium concentration and liver temperature, and ensure that the liver is adequately oxygenated.

### 3.3. Micro Fluidic Devices

Another emerging field in the study of the metabolism of hepatocytes is the use of micro fluidic devices to create liver-like physical structures for hepatocytes, or to assess hepatocyte function on an individual level, although a stoichiometric-based analysis for hepatocytes in micro fluidic devices has not been reported yet. The advantages of micro fluidic studies are the greatly increased control over cell position, cell neighbors, and the amount of medium that a cell is exposed to [51]. These provide strong incentives for investigators studying hepatocytes, where the control of cell position and medium allows for better *in vivo*-like conditions, and better exposure to substrate. For example, Yamada *et al*. were able to use a size exclusion micro fluidic device in order to separate hepatocytes from other cell types that are native to the liver [52], allowing for pure hepatocyte studies that do not risk the compromise of cell viability due to centrifugation and other current separation methods. Micro fluidics have also been used to assess metabolic and protein activity on a layer-by-layer basis, allowing for a dynamic view of the way hepatocytes behave in a three-dimensional, parallel system [53]. Cell cultures are currently unable to assess this, as the metabolic data that is generated is assessed in bulk in medium concentrations. Micro fluidic cultures of hepatocytes have also been successful at mimicking *in vivo* conditions by forming bile cannuli, which are an important part of liver morphology that has not been captured in cell culture studies [54]. One of the largest challenges in metabolic studies of hepatocytes is the determination of intracellular concentrations of substrates, but Zhao *et al*. have been able to create a micro fluidic system that can assess intracellular metabolite concentrations at a single cell level, which could significantly improve the computational accuracy of metabolic models in the future [55]. Though micro fluidic devices generally include only hepatocytes, efforts have been made to create more *in vivo*-like conditions by the incorporation of fibroblasts and other mammalian cell types into the system [56]. Normally, the experimental determination of intracellular concentrations within hepatocytes requires both the labeling of substrates of interest, and the quenching of metabolic reactions at the time of analysis [57]. One of the advantages, therefore, of these novel microfluidic devices, is that due to the rapid speed of cell lysing and separation, quenching is not required, and intracellular concentrations of amino acids can be directly assessed. Lee *et al*. have created an artificial barrier in a micro fluidic device with properties similar to endothelial cells in order to assess the effects of the endothelial barrier on hepatocyte function [58]. Overall, the field of micro fluidics is not as developed as cell culturing and IPRL systems. However, it is a potent technique for assessing qualities that are difficult to measure in a bulk scale, and for fine tuning the experimental control of hepatocyte environments. 

## 4. Stoichiometric Models for Hepatic Networks

### 4.1. Hepatic Network Construction

Metabolic network analysis integrating multiple reaction rates to analyze the network and cell behaviors have been increasingly applied for liver systems and these studies are becoming important for physiological and topological characterization of the liver at cellular or organ level. Therefore, the structure of the liver network, *i.e.*, liver-specific biochemical reactions and pathways, should be first well described. However, constructing a metabolic network requires collection of a variety of genomic, biochemical, and physiological data from the primary literature and databases. The liver metabolic network was originally developed for perfused livers and hepatocyte cultures [1,5,6,59]. It has already been shown that there is a good consistency between the measurements and assumed metabolic pathways of the model in these studies. Given the physiological properties of the liver and experimental conditions, the network involves all major liver-specific pathways, including central carbon and nitrogen metabolism, such as gluconeogenesis (or glycolysis, based on the fed/fasted state of animals), urea cycle, fatty acid metabolism, pentose phosphate pathway, TCA cycle, glycogen metabolism and amino acid metabolism. In most of the previous studies, especially in liver perfusion studies, animal models have typically been fasted prior to performing liver perfusions in order to simplify metabolic network analysis [1,2,3,4,5,6]. In other words, under these conditions, certain simplifying assumptions can be used, such as lack of glycogen storage and inhibition of all strictly glycolytic enzymes. These assumptions stem from the fact that during post absorptive states, the liver often provides energy to the rest of the body through significant gluconeogenesis and glycogen breakdown [24]. In some instances, it was assumed that the glycolytic and gluconeogenic states are mutually exclusive; therefore, different metabolic models including either the gluconeogenic or glycolytic pathways were utilized. Both models were then fitted to the experimental data and the best fitting model was used to analyze the fluxes [59,60]. Since a “unified” metabolic flux analysis approach that is not limited to the fasted state, but rather that could be similarly applied to both fed and fasted states is required, Orman *et al*. modified the previously published network to simultaneously include both glycolytic and gluconeogenic pathways, fatty acid synthesis and oxidation, as well as glycogenesis and glycogenolysis [46].

The hepatic model discussed above is a medium scale network. Very recently, genome scale hepatic models have been developed for the analysis of liver physiology. Gille *et al*. [61] constructed a comprehensive metabolic network of the human hepatocyte by using global human metabolic network and manually evaluating more than 1500 articles, reviews and biochemical textbooks. The network consists of 777 metabolites in six intracellular and two extracellular compartments and 2539 reactions, including 1466 transport reactions. They rigorously tested network functionality by means of constraint-based modeling techniques, *i.e.*, flux balance analysis. Jerby *et al*. [62] also developed a genome-scale hepatic model and investigated its ability to carry out hepatic metabolic functions. Integrating different tissue specific data sources, such as literature-based knowledge, transcriptomic, proteomic, metabolomic and phenotypic data, they proposed an algorithm generating a tissue specific model from the generic human model. The final hepatic model that they constructed includes 1827 reactions and 1360 metabolites.

### 4.2. Steady State Flux Analysis

Generally, metabolic flux analysis (MFA) has been used to characterize the hepatic intracellular reactions by using a set of measured extracellular fluxes and the hepatic metabolic network that has been constructed [1,2,3,4,5,6,59]. MFA is based on mass balances of internal metabolites. Since it is assumed that the accumulation of internal metabolites is insignificant, which implies the pseudo steady state, the mass balance is written as follows: 


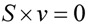
(1)


where **v** is the flux distribution vector, and **S** is the stoichiometric matrix where rows correspond to the metabolites and columns represent the reaction rates. Using a set of measured fluxes (**v_m_**), Equation 1 is rewritten as below in order to calculate the unknown fluxes (**v_u_**): 


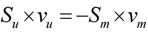
(2)

In MFA analysis, the system described by Equation 2 should be determined in order to identify unknown fluxes uniquely, *i.e.*, the number of linearly independent equations should be equal to number of unknown fluxes. In the studies previously published [1,2,3,4,5,6,59], the metabolic network models are generally over-determined since the number of measured extracellular fluxes is larger than the rank of the stoichiometric matrix. In this case, the unknown fluxes were calculated by minimizing the sum of square errors between the measured and the estimated fluxes. Given that the system was over-determined, the presence of measurement errors and consistency of the metabolic network has been also evaluated by using statistical tests in these studies.

In some instances, the system described by Equation 2 might be underdetermined, *i.e.*, the number of experimental measurements is not enough to determine the unknown fluxes uniquely. Flux balance analysis (FBA) is one of the metabolic engineering tools widely applied to solve this problem. FBA is based on an optimization problem with an objective function depending on the physiological or topological properties of the systems, and with the well-known constraints, including mass balance equations, reaction reversibility or thermodynamic constraints. The steady state solution space defined by linear equations and inequalities is called the flux cone. The optimum point within this cone, which is the solution of the problem, is described by the objective function. However, proposing an appropriate objective function for liver systems is a major challenge, since liver cells exhibit various phenotypic properties depending on the environmental conditions. Although some objective functions such as maximization of cell growth, or maximization of ATP production rates, have been commonly used for microbial systems, these phenotypes might not be associated with liver systems. A number of studies identified the intracellular hepatic fluxes by minimizing the error between the observed and calculated external fluxes [63,64,65]. This is quite reasonable since experimental measurements or external fluxes are related to internal reactions. The prediction error (which is minimized) should be normalized in order to prevent flux estimation biased towards the measured fluxes with large values (
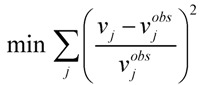
, where *v_j_* and *v_j_^obs^* are the predicted and observed external flux values, respectively). Another objective function that has also been used for hepatocytes is maximizing the summation of weighted fluxes (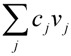, where the coefficient *c_j_* is the weight of flux *v_j_*). Therefore, a weight can be attributed for each flux, which shows the importance of that flux. Yang *et al*. [66] and Nolan *et al*. [63] applied a bi-level optimization problem for the hepatic network where upper level objective function minimizes the relative error, whereas the lower level maximizes the summation of weighted fluxes. They solved the bi-level program by reformulating it into a single-level program using the Karush-Kuhn-Tucker or the primal-dual strategies. Furthermore, Uygun and co-workers [65] formulated a three-level optimization problem in order to determine a minimum set of fluxes that are of major importance to the liver cells while minimizing the relative error and maximizing the summation of weighted fluxes. They solved this mixed integer nonlinear programming problem (MINLP) in an iterative scheme until sufficient prediction accuracy had been obtained. By this data-mining procedure, they aimed to identify possible metabolic objectives for hepatocytes cultured *in vitro*.

There are other objective functions that have been used for liver systems. These objective functions are generally based on the physiological properties of the liver. Calik and Akbay [67] calculated flux distributions in the fibrotic and healthy liver cells by maximizing respectively the collagen and palmitate synthesis. Yang *et al*. [68] used a FBA model where the urea output had been maximized in order to optimize amino acid supplementation and hormone levels in the medium. The aim is to increase the performance of the liver-specific function, *i.e.*, urea production, by designing a rational medium composition. Due to the possibility of alternative flux distributions resulting in the same optimum value, they applied mixed integer programming to calculate all solutions. In fact, hepatocytes exhibit multiple functions that need to be considered simultaneously. Yang and co-workers further investigated the effects of amino acid supplementation on the objectives of urea production and fatty acid oxidation in hepatocytes [69]. The model with multi-objectives they utilized also included the constraints that represent the transport limitations of amino acids. They solved the problem by maximizing the primary objective, *i.e.*, urea production, while converting the other objective (fatty acid oxidation) into a constraint. Actually, choosing different values for the constraint describing the rate of fatty acid oxidation determined the Pareto optimal solutions where urea flux and fatty acid oxidation rate are best compromised. Sharma *et al*. [70], on the other hand, investigated Pareto optimal set of solutions corresponding to liver-specific functions of urea and albumin secretion in order to analyze the importance of amino acids in the supplementation. Nagrath and co-workers [71,72] also developed a multi-objective optimization approach to characterize the Pareto frontiers in gluconeogenic and glycolytic hepatocytes for various combinations of some global or liver-specific objectives (albumin synthesis, glutathione synthesis, NADPH synthesis, ATP generation, and urea secretion).

Metabolic pathway analysis (MPA), mainly consisting of elementary modes and extreme pathways, has been recently used to characterize the liver networks. Elementary modes consist of the minimum number of reactions that exist as a functional unit. Extreme pathways that correspond to extreme rays of flux cone are the independent subset of elementary modes. However, analyzing a biological system through a set of extreme pathways can result in the exclusion of possibly important modes [73,74]. Pathways which only rely on the stoichiometry of the network can be calculated using the algorithm provided by CellNetAnalyzer [75], which is the further development of *FluxAnalyzer* [76]. Pathway analysis has turned out to be very important tool since it can identify all possible paths of the external metabolites and the contribution of the pathways to the flux distribution. In other words, a flux distribution vector can be described by a linear combination of the elementary modes, or extreme pathways, thus a weight can be assigned to each corresponding pathway (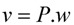, where *w* denotes a vector involving the weight for each elementary mode; and *P* is the matrix of elementary modes). However, the decomposition of a steady state flux vector into pathways is not always unique for large networks because the number of pathways is not usually equal to the dimension of the null space of the stoichiometric matrix [77]. To overcome this problem, different objective functions have been proposed in the literature, such as maximization of the number of elementary modes, minimization of the elementary mode activity and the entropy maximization principle [78,79,80,81]. Orman *et al*. further introduced other optimization algorithms, including maximization of activity of short pathways and maximization of activity of liver-specific pathways, including urea and glucose production, to analyze the hepatic metabolism with the data sets obtained from perfused livers of fasted rats receiving burn injury [64].

Thermodynamic-based metabolic flux analysis has been profoundly analyzed in microbial systems [82,83]. Since an exergonic reaction can be a driving-force for an endergonic reaction if these two reactions are coupled in the same pathway, elementary modes were also used to formulate thermodynamic feasibility constraints for hepatic metabolic network to reduce the feasible range of intracellular fluxes [63,66,84,85,86,87]. The Gibbs free energy of the pathway is the summation of the Gibbs free energies of reactions involved in that pathway which should be less than or equal to zero. Given the weight values of pathways, the pathway-based thermodynamic constraint could be further modified to eliminate the thermodynamically infeasible pathways: 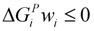, where 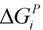 and *w_i_* are respectively Gibbs free energy and weight value of the pathway *i* [87]. This condition ensures that the pathway *i* can be active (w*_i_* ≥ 0) if its Gibbs free energy is less than zero (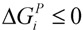), otherwise it is not active (w*_i_* = 0).

The hepatic metabolic network includes both glycolytic and gluconeogenic pathways, fatty acid synthesis and oxidation, as well as glycogenesis and glycogenolysis [87]. Therefore, this might result in potential futile cycles due to the reaction pairs involved in these pathways (such as glucokinase and glucose-6-phosphatase). When two metabolic reactions or pathways in opposite directions are active simultaneously, a futile cycle is formed. Without an additional constraint, the mathematical model might assign physiologically irrelevant flux values for these reaction pairs. This can be prevented by directly measuring the cyclic fluxes using isotope tracer, or including the mass balances of energy metabolites such as ATP, NADH, *etc.*, if they are associated with cyclic reactions. However, the latter requires a complete metabolic network. In general, reciprocal control between the enzymes forming a futile cycle inhibits its formation. This is reasonable, since futile cycles result in energy waste. We recently proposed a mathematical formulation by introducing binary variables to prevent futile cycles in liver systems [87]:


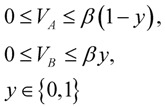
(3)

This constraint described by the Equation 3 allows the two reactions (V_A_ and V_B_ which form a futile cycle) to inhibit each other. *y* is a binary variable, and β is a very large number which forces *y* to be 0 (thus V_B_ is 0) if the reaction V_A_ is active. If the reaction V_A_ is zero, then V_B_ can take any value.

## 5. Applications and Major Outcomes of Stoichiometry-Based Hepatic Models

Flux analysis approaches have been increasingly applied for liver systems to characterize the liver functions and objectives, to improve the functions of cultured liver cells or perfused livers and to analyze the hepatic metabolic alterations caused by various disease states, mainly by burns and infections (Table 1). Moreover, liver systems were also used to characterize the toxic effects of drugs and to provide some cues to rationally design nutritional therapies or drugs to alleviate the metabolic effects of diseases and injures. These have been explained in great detail in subsequent sections. 

**Table 1:** Published studies where steady state hepatic fluxes have been determined. FBA: Flux balance analysis, MPA: Metabolic pathway analysis, MFA: Metabolic flux analysis.

Aim	Method	References
To investigate the metabolic objectives of cultured hepatocytes.	FBA	Uygun *et al*. [65]
To analyze the liver metabolism under fasted state	FBA	Orman *et al*. [7]
To analyze the liver metabolism under fasted state	FBA, MPA	Orman *et al*. [87]
To identify the metabolic changes associated with the cytotoxicity of saturated free fatty acids	MFA	Srivastava and Chan [88]
To design an optimum amino acid supplementation to increase the liver functions (computational)	FBA	Sharma *et al*. [70]
To design an optimum amino acid supplementation to increase the liver functions (computational and empirical)	FBA	Yang *et al*. [66,68,69]
To improve the hepatic functions with insulin, amino acid and plasma supplementation	MFA	Chan *et al*. [59,89]
To improve the perfused livers’ functions with red blood cells	FBA, MPA	Orman *et al*. [46]
To analyze the effects of burn injury on the flux distribution of liver metabolism	MFA	Lee *et al*. [5,6]
To determine hepatic flux changes caused by burn and septic shocks	MFA	Banta *et al*. [1]
To investigate the D-Galactosamine induced rat liver failure	MFA	Arai *et al*. [4]
To investigate D-Galactosamine induced rat liver failure (fluxes were measured at multiple time points)	MFA	Yokoyama *et al*. [3]
To investigate the effects of glucose and insulin on hepatic carcinoma cells	FBA	Iyer *et al*. [85]
To analyze the therapeutic effects of Dehydroepiandrosterone	MFA	Banta *et al*. [2]
To investigate the effects of hepatotoxic compounds on liver metabolism	MFA	Niklas *et al*. [90]
To analyze the effects of triadimefon on the primary rat hepatocytes	FBA	Iyer *et al*. [84]

### 5.1. Characterization of Liver Functions and Objectives

One of the reasons of application of stoichiometric analyses for liver systems is to understand the physiology of the liver and characterize the hepatic metabolic network. Using flux balance model, Uygun *et al*. investigated the possible metabolic objectives for hepatocytes cultured *in vitro* [65]. They found that *in vitro*-cultured hepatocytes maximize oxygen uptake, coupling of urea and TCA cycles and synthesis of serine and urea. Orman and co-workers used a flux variability approach, sampling analysis and singular value decomposition to analyze the metabolic network for perfused liver under fed and fasted conditions [7]. They observed that fasting mainly affects glycolytic and gluconeogenic reactions, aspartate and glutamate metabolism and PPP. They showed that protein metabolism accounts for a small portion of the nitrogen metabolism. Using the same data set, the same group further characterized the hepatic network under fasted state by exploring a methodology based on elementary mode analysis, considering thermodynamic constraints and enzymatic regulatory properties for the formation of futile cycles [87]. The resultant mixed integer quadratic programming identified that gluconeogenesis, glycogenolysis and fatty acid oxidation were active in both fasted and fed states. It was shown that fasting increased the fluxes in gluconeogenic reactions whereas it decreased fluxes associated with glycogenolysis, TCA cycle, fatty acid oxidation and electron transport reactions.

### 5.2. Improving *in Vitro* Liver Functions

Liver cells are significantly used in research programs to characterize not only the hepatic metabolic functions but also hepatic signaling and genetic functions. They are also used for medicinally important devices such as the bioartificial liver, which is one of the promising technologies for the treatment of liver failure. However, hepatocytes exhibit poor liver specific functions in *in vitro* conditions, which is very critical for the aforementioned studies. Sharma and co-workers analyzed the importance of amino acids in the supplementation by using a flux balance approach in order to increase the functions of urea and albumin secretion [70]. Similarly, Yang *et al*. used FBA approaches to design an optimum amino acid supplementation [66,68,69]. They experimentally verified that urea and albumin production under the designed amino acid supplementation was found to be increased compared with previously reported (empirical) amino acid supplementation [59,89]. Chan *et al*. used MFA analysis to elucidate the changes in intracellular hepatic fluxes following a low insulin pre-conditioning and plasma exposure with amino acid supplementations [59,89]. They observed that a low level of insulin decreased the clearance of glucose and glycerol. Subsequent plasma exposure with amino acid supplementation augmented gluconeogenic pathways and fatty acid oxidation. The same group further studied the effects of both hormone (insulin and hydrocortisone) and amino acid supplementation on hepatic functions. It was identified that *β*-oxidation, tricarboxylic acid (TCA) cycle fluxes and gluconeogenic fluxes were up-regulated by both hormone and amino acid supplementation.

Isolated liver perfusion systems have been commonly used to characterize intrinsic metabolic changes in liver under various conditions. In order to improve the perfusion system, Orman *et al*. [46] used a flux balance approach based on elementary mode analysis to compare the effects of three modes of oxygen delivery to perfused livers. These are normoxic (arterial) perfusate (21% O_2_), hyperoxic perfusate (95% O_2_), and hyperoxic perfusate with oxygen carriers (95% O_2_ + 10% hematocrit using bovine red blood cells). They found that perfused livers consumed oxygen up to the rate of 400 μmole/g liver/h when the perfusate contained red blood cells (RBCs). On the other hand, using 95% O_2_, in the absence of oxygen carriers, oxygen uptake was significantly reduced; urea and ketone body production were significantly decreased, and metabolic pathway analysis elucidated that significant anaerobic glycolysis occurred.

### 5.3. Characterization of Disease-Related Changes in the Liver

Liver is a critical organ in mammalian physiology, serving to synthesize proteins both constitutively and in the acute phase, as well as regulate whole body energy levels through metabolic pathways and signaling molecules. The liver’s roles in the Cori cycle and the urea cycle are also well known functions. Therefore, it is necessary to understand the changes that the liver undergoes in response to injury, and the underlying regulations that govern those changes, in order to fully understand the impact of the injury. Many studies have shown that significant metabolic alterations take place in the liver in response to various injuries, including sepsis and burns. The liver perfusion system creates an important portal to characterize these alterations. Lee and co-workers used metabolic flux analysis to determine the effects of burn injury on the flux distribution of liver metabolism [5,6]. They showed that the fluxes in mitochondrial electron transports, the TCA and urea cycles, gluconeogenesis, and the pentose phosphate pathway significantly increased post-burn on day 4 in the rat liver [5]. They also profiled the dynamic changes of fluxes in the rat livers up to 7 days after burn injury [6]. They observed that burn injury sequentially up-regulated the urea cycle, the PPP and the TCA cycle. Banta and co-workers [1] analyzed the metabolic fluxes in rat livers after burn and cecum ligation and puncture (CLP). The results showed that both burn and CLP resulted in the most dramatic changes in the intracellular fluxes. The D-Galactosamine induced rat liver failure model has been also studied for a comprehensive understanding of the hepatic metabolic pathways affected by fulminant hepatic failure (FHF). Arai *et al*. showed that D-Galactosamine inhibited hepatic glucose synthesis, due to the reduction in amino acid entry into TCA cycle [4]. Yokoyama and co-workers profiled the metabolic fluxes in rat liver, considering different time points (1, 4, 8, 12 h) following the D-galactosamine treatment [3]. They showed that gluconeogenesis, TCA cycle and mitochondrial electron transport fluxes were decreased just after the D-galactosamine injection. They also demonstrated that gluconeogenesis had switched to glycolysis after around 8 h while a net release of many amino acids was observed around 12 h following the D-galactosamine treatment.

Iyer *et al*. characterized the hepatic carcinoma cells cultured under various levels of glucose and insulin up to five days [85]. Interestingly, it was elucidated that glucose consumption was greater for low glucose medium compared to high glucose medium and urea productivity was highest in glucose-free medium. They showed that in high and low glucose media, glycolysis, glutaminolysis and oxidative phosphorylation were the main sources of energy, and the presence of insulin decreased glycerol uptake rate and the fluxes involved in lipid metabolism, independent of glucose concentration.

### 5.4. Proposing Treatment Techniques and Analyzing Effects of Drugs

As emphasized previously, it is necessary to understand the underpinning dynamics of the liver response (or liver failure) during the disease conditions. This also provides insights for better treatments in clinical settings. Using pathway analysis, Orman and co-workers characterized the hepatic metabolism following the burn injury [64]. They identified the main sources for the production of certain products (especially urea and glucose) which are up-regulated during the hyper-metabolic state. A set of metabolites, including glutamine, arginine and aspartate was found to be important, which might be utilized as nutritional supplement for the burn patients to manipulate biochemical environment of the hyper-metabolic liver in order to reduce physiologic stress caused by burn injury. A very important study published by Banta and co-workers explored the effects of Dehydroepiandrosterone (DHEA), used as a treatment for trauma patients, on hepatic metabolism following burn injury [2]. Their metabolic flux analysis showed that DHEA administration appeared to normalize hepatocellular metabolism in burned rats but decreased the PPP flux. They speculated that the liver’s ability to recycle endogenous antioxidants was impaired after the burn injury. 

Subtoxic effects of drugs and other medical products were also tested on the hepatocytes, the primary cells having a function of detoxification. Niklas *et al*. [90] analyzed the effects of three different hepatotoxic compounds (amiodarone, diclofenac and tacrine) on liver metabolism. They found that diclofenac and tacrine increased the TCA-cycle. Iyer *et al*. [84] studied the effects of triadimefon, a tumorigenic conazole, on the primary rat hepatocytes. Their FBA demonstrated a switch from fatty acid synthesis to fatty acid oxidation in cells exposed to triadimefon. They interpreted that fatty acid oxidation might be one of the important energy sources required for triadimefon detoxification.

## 6. Current Challenges

Liver metabolism is very complex, and constructing a hepatic metabolic network requires intensive experimental evidence. Most of the hepatic metabolic network studies for perfused livers and hepatocytes cultures have utilized a medium scale network, including the major liver specific reactions or pathways. It has been already shown that there is a good consistency between the network and measured fluxes [5,6]. Although genome scale hepatic networks have been recently published, it is still very difficult to apply metabolic engineering tools, including MFA, FBA and MPA, for these large scale networks. To obtain a physiologically relevant flux distribution, MFA requires hundreds of flux measurements, which is experimentally very difficult and expensive. Although it is possible to calculate a flux distribution vector for larger networks using FBA, considering an objective function and various constraints, it might be impossible to validate the optimal flux distribution described by the objective function. Moreover, algorithms that are more robust are required to identify the extreme pathways or elementary modes for larger networks, since the number of pathways for larger networks might be countless. Another important problem which might prevent the application of metabolic engineering tools for the liver systems is that the objective functions used in this type of analysis of liver are not well characterized. The fluxes determined by the objective functions would be correct only in very specific conditions which might not occur in perfused livers or cultured cells. The liver has a very robust metabolic network affected by metabolites and hormones; therefore, it exhibits different phenotypes depending on the environmental conditions. Different objective functions based on the topological and physiological properties of the liver have been used in the literature [64,67,68]. Focusing on consistent results predicted by different objective functions might reveal potentially important properties of liver metabolism [64].

Although the aforementioned methods provide a comprehensive map of stationary flux distribution in the metabolic network, they clearly fall short of giving insight into metabolic regulation. Moreover, the steady state assumption for the liver might not be valid since it permanently switches between glucose consumption and glucose production, triglyceride formation and triglyceride degradations, *etc*. Most of the studies have been performed *in vitro* without exposing the liver to the hormones or signaling proteins that might drastically affect these metabolic switches. Inherent regulatory mechanisms that might also result in changes in hepatic fluxes were also ignored in published studies. The condition for the pseudo-steady-state approximation for metabolic flux balance is that the rate of change of (internal) metabolite concentration should be small compared to its rate of turnover (flux). It has been shown that the fluxes are considerably higher than the actual changes in the intracellular metabolite concentration in HepG2-C3A cells, which supports the pseudo-steady-state approximation [88].

Liver perfusion experiments are performed in a very short period of time, *i.e.*, the isolated liver is exposed to a defined media (perfusate) for about 1 h. The perfused livers are not exposed to circulating factors (e.g., insulin, glucagon and other hormones) or substrate concentrations changes that occur *in vivo*. It is likely that switches between the major pathways in perfused livers do not occur during the course of experiment. Since the concentrations of metabolites in the perfusate reservoir were found to change linearly as a function of time (*i.e.*, constant metabolite production or consumption rates) [1,2,3,4,5,6,7], implying that the perfused liver was metabolically stable for the duration of the perfusion, the pseudo steady-state assumption is reasonably valid. However, for hepatocytes cultured *in vitro*, flux measurements have been performed every 24 h [66,67,68,69,84]. Therefore, these studies do not guarantee whether or not metabolic switches between the pathways in hepatocytes take place within a 24 h period.

Despite the significant advances that have been made in recent years with regards to metabolic flux analysis in the liver, there is still significant room for improvement in many areas of the field. Although mathematical models are becoming better structured and optimized with the inclusion of multiple optimization parameters and added physiological and thermodynamic constraints, investigators are still left with the difficult task of predicting the overall goal of the liver. Because of the difficulty involved in obtaining intracellular metabolite concentrations, the resulting underdetermined system can only be solved through an educated guess at the objectives that result in the measured fluxes [77]. Thus, there is great potential in the field for the development of new methods to measure intracellular concentrations, using micro fluidic devices instead of the significantly more time-consuming radio labeling studies that have been performed in the past [55]. While the current devices are able to analyze the contents of a single cell in the micro fluidic device, it is, in theory, possible to construct a device capable of measuring many intracellular concentrations at once through a parallel set up, which could provide a much more complete overview of intracellular metabolism. Although an entirely specified system would allow investigators to simply solve for the unknown fluxes, optimization techniques will be needed in order to understand the underlying mechanisms that give rise to observed fluxes. The mechanisms that regulate various enzymatic proteins within the liver can each be considered to be objective functions that are controlled and altered by various stimuli. Refined optimization models already hold the ability to incorporate a variety of constraints [64], and the ability to apply these models to increasingly specified systems will allow investigators to mathematically assess not only the alterations in metabolism, but the changes in the liver’s biological objective function, which is critical to injury care. Hypermetabolism is defined as a series of changes within metabolism that lead to an up-regulation of energy production, urea production and, on a more systemic level, muscle wasting and serum protein loss [91]. By identifying the underlying objective function(s) that define hypermetabolism, and contrasting them with those present in normal liver function, the way is paved for the identification of pathways and proteins for therapeutic treatment that will reset those objective functions to normal, non-pathophysiological values.

Although experimental procedures developed for liver cell cultures or perfused livers have provided important information regarding the liver metabolic properties, an obvious drawback is the artificial *ex vivo* environment that may potentially induce aberrant metabolic patterns. In particular, in perfusion systems it is quite challenging to mimic the physiological environment (pH, temperature, flow rate, adequate oxygenation, *etc*.) of the liver that is isolated from the body. Among these parameters mentioned above, inadequate oxygenation could by itself alter gene expression level and promote anaerobic pathways [46]. However, the majority of liver perfusion studies did not utilize oxygen carriers. Orman *et al*. have recently showed that hyperoxic oxygenation without the use of oxygen carriers was not sufficient to support perfused rat liver function [46]. The use of red blood cells in the IPRL system was able to alleviate hypoxia in the tissue, and allow for a more physiologically relevant response, but there is still room for improvement in the architecture of the experiment. The composition of the medium is extremely important in IPRL experiments as well [48]. However, there is little known about the effect that the absence (or presence) of various serum proteins will have on liver metabolism. It has been shown by previous studies that uninjured animals have a cytokine profile which contrasts significantly with their injury profile [92], however perfusion experiments that have been conducted in the last twenty years have not considered the inclusion of a baseline protein cocktail that mimics *in vivo* conditions. The challenge therein is threefold: Firstly, the baseline dynamics of serum proteins in the absence of injury must be characterized. The presence of the circadian rhythm, which is a time-based gene regulation system that modulates metabolism based on feeding and sleeping cycles [93], implies that there is the potential for variation in serum protein concentrations with time. Unfortunately, many proteins that exist in serum have short half lives [94], and so the next challenge involved in this evolution of the IPRL system requires the development of methods that can reliably synthesize these proteins either on site, or in ways that allow them to be stored and remain viable. Already efforts are being made to extend protein half life [95], therefore finding ways to import those technologies and adapt them for serum protein synthesis is an upcoming challenge for investigators. The final challenge in the development of this extension of the IPRL system is the characterization of the effects that these proteins have on the liver system during experiments. Though it could be argued that no protein would be present in serum without a purpose, it is possible that not all proteins are intended to enact metabolic changes on the liver, and thus a characterization of these effects, both in isolation, and in synergy, is necessary to identify proteins that are necessary in order to move the IPRL system further toward true *in vivo* conditions. This also has the added benefit of increasing understanding of liver metabolism, both how it changes during circadian rhythms, and which proteins are responsible for its dynamics. Since circadian rhythms have been associated with immune regulation, and their disruption with pathophysiology [96], these studies would have dual relevance.

One other challenge that *in vitro* studies encounter when performing experiments on primary hepatocytes is the phenomenon of hepatocyte zonation. Although other cell types that reside within the liver also manifest functional differences based on their location [97], the zonation behavior of primary hepatocytes can affect the results of *in vitro* metabolic studies by utilizing metabolic pathways that may not be consistent with the rest of the liver’s behavior. The concepts of metabolic zonation are critical to the design of *in vitro* primary hepatocyte studies that aim to assess metabolic function, as an imbalance between metabolic states within the cell culture may skew the results toward non-physiological behavior. The use of oxygen gradients in hepatocyte cell cultures can produce hepatocyte zonation, similar to what has been observed in *in vivo* studies [98]. A viable alternative to generating a gradient is to simply simulate the behaviors of perivenous and periportal hepatocytes separately, by exposing cultures to varying levels of oxygen [99]. Primary cultures of two different hepatocyte types can be generated using a digitonin-collagenase method [100], which first destroys the periportal hepatocytes, and then isolates the perivenous cells. Retrograde digitonin infusions can then be used to isolate periportal cells, allowing for *in vitro* experiments to observe metabolic changes in each zone independently. A more *in vivo* approach to differentiating the functions of the perivenous and periportal zones are to use ortho- and retrograde liver perfusions that contain labeled substrates [101], and follow the differences in uptake and metabolism at each zone of the liver. By accounting for hepatocyte zonation in *in vitro* studies, it is possible for investigators to gain further insight into physiological behaviors of the whole liver, while also potentially generating additional constraints for metabolic models. 

Finally, there is still room for improvement in the area of cultured hepatocytes, and the methods by which investigators are attempting to generate *in vivo* conditions. Unlike the IPRL systems, there is a significant amount of control over the environment of hepatocytes in culture, and thus improvements on the system would be of immense benefit to the collective understanding of liver metabolism. Efforts have already been made to create culture systems in a plated environment that mimic *in vivo* conditions [37,38], as well as in micro fluidic systems, which are able to mimic the physical properties of the liver [58,102]. However, although each of these improvements individually is able to make the hepatocyte environment similar to *in vivo* conditions, it will likely require a combination of effects (adipocyte matrix, bile cannuli and an endothelial barrier, for example) to truly be able to mimic the behavior of *in vivo* hepatocytes. Thus, the challenge that remains for investigators working with hepatocyte cell cultures is to incorporate and consolidate the various new methods being developed that mimic *in vivo* conditions. There is a significant technical challenge involved in reconciling techniques that has been applied to cell cultures to a micro fluidic device, and *vice versa.* However, just as the liver does not contain just an epithelial barrier or bile cannaliculi, but rather both simultaneously, an appropriate *in vitro* model must also contain both of these elements. This challenge, combined with the need for better medium control in IPRL systems, and the development of better techniques for intracellular flux determination and modeling, are necessary for the development and enhancement of the understanding of metabolic flux and liver function, both on a computational and biological level. 

## 7. Conclusions

The liver is an important organ, having a very complex metabolic network. Metabolic engineering tools and advanced experimental methods have been applied to this complex system to characterize the underlying mechanisms of various conditions leading to metabolic alterations in the liver. With new advanced methodologies, more insight will be gained regarding the liver metabolic network properties and liver associated diseases. In this review, we mainly focused on the published studies where stoichiometric models were utilized for liver systems. The experimental and computational methods as well as current challenges have been extensively discussed.
